# Research progress of diabetic osteoporosis: a comprehensive review

**DOI:** 10.3389/fendo.2025.1595228

**Published:** 2025-09-02

**Authors:** Xun Ma, Xiaoqian Zhang

**Affiliations:** ^1^ School of Clinical Medicine, Shandong Second Medical University, Weifang, China; ^2^ Department of Endocrinology and Metabology, The First Affiliated Hospital of Shandong First Medical University & Shandong Provincial Qianfoshan Hospital, Shandong Institute of Nephrology, Jinan, China

**Keywords:** diabetic osteoporosis, pathophysiological mechanisms, glucose-lowering agents, anti-osteoporotic agents, traditional Chinese medicine

## Abstract

Diabetic osteoporosis (DOP) is a complex metabolic bone disorder characterized by impaired bone quality and increased fracture risk in patients with diabetes mellitus. The interplay between hyperglycemia, insulin resistance, and bone metabolism underscores the need for integrated therapeutic strategies that address both glycemic control and bone health. This review systematically examines the molecular mechanisms of glucose-lowering and bone-protective agents, highlighting their dual roles in managing DOP. We discuss the pathophysiological pathways underlying DOP, including insulin/IGF-1 deficiency, advanced glycation end products (AGEs) accumulation, oxidative stress, and vascular damage. Furthermore, we explore the mechanisms of action of antidiabetic drugs (e.g., metformin, GLP - 1 receptor agonists, SGLT2 inhibitors) and anti-osteoporotic agents (e.g., bisphosphonates, teriparatide, strontium ranelate), emphasizing their potential synergies and risks. Finally, we outline future directions for developing novel therapeutics and optimizing combination therapies to achieve dual metabolic and skeletal benefits in DOP patients.

## 1 Introduction

Diabetes and osteoporosis are globally prevalent chronic metabolic diseases whose pathological interplay in diabetic osteoporosis (DOP) poses a critical clinical concern. According to the International Diabetes Federation (IDF), approximately 537 million individuals aged 20 – 79 years were diagnosed with diabetes in 2021, a figure projected to rise to 783 million by 2045, with type 2 diabetes (T2DM) accounting for over 95% of cases. Concurrently, OP affects 19.7% of the global population, causing more than 8.9 million fractures annually, with hip fracture incidence expected to double by 2050 (1, 2). Epidemiological studies reveal that 50 – 66% of diabetic patients exhibit reduced bone mineral density (BMD), of whom 33% are diagnosed with DOP, underscoring its clinical significance as a major diabetic complication (3, 4). Despite normal or high BMD, T2DM patients face 40 - 70% higher fracture risk than non-diabetics, highlighting bone quality impairment and microstructural damage—not just bone loss—as key DOP characteristics (5, 6).

The pathophysiology of DOP involves multifaceted interactions. Chronic high blood sugar disrupts bone balance by causing oxidative stress, AGEs buildup, and inflammation, which weaken bone formation while increasing breakdown (7, 8). New research shows blood sugar fluctuations harm bone blood vessels and bone cells more than constant high sugar levels, mainly through oxidative stress, mitochondrial damage, and iron-related cell death (9). Ferroptosis, an iron-dependent lipid peroxidation-driven cell death mechanism, is amplified by glucolipotoxicity in T2DM, contributing to osteoblast dysfunction and osteocyte loss (10, 11). Furthermore, dysregulated autophagy (particularly mitophagy) and vitamin D receptor (VDR) gene polymorphisms (e.g., TaqI, EcoRV) impair skeletal homeostasis, while leptin and other adipokines secreted by perirenal adipose tissue exacerbate bone remodeling imbalance via pro-inflammatory pathways (12–14). Impaired osteogenic differentiation of bone marrow mesenchymal stem cells (BMSCs) and aberrant expression of key genes (e.g., FOXQ1) further underpin DOP pathogenesis (15– 16).

Emerging research emphasizes the dual mechanistic roles of glucose-lowering and anti-osteoporotic therapies. While conventional antidiabetic agents improve glycemic control, certain drugs (e.g., thiazolidinediones) may exacerbate bone loss by inhibiting osteoblast differentiation. In contrast, newer agents (e.g., GLP - 1 receptor agonists, SGLT2 inhibitors) demonstrate potential bone-protective effects via metabolic and anti-inflammatory modulation. Anti-resorptive therapies (e.g., bisphosphonates, RANKL inhibitors), though effective in suppressing bone resorption, exhibit variable efficacy in diabetic populations due to glucose metabolism dysregulation and vascular complications. Crucially, no comprehensive review has systematically integrated the pathophysiological interplay between these therapeutic classes in DOP management. This gap hinders the development of synergistic strategies to address concurrent glycemic and skeletal dysregulation.

In summary, the prevention and treatment strategies for DOP must be grounded in its multifactorial pathological mechanisms, with an emphasis on exploring individualized therapeutic targets. We systematically searched PubMed, Web of Science, and Embase (2018 – 2024) using MeSH terms: (diabetic osteoporosis OR diabetes bone disease) AND (pathogenesis OR drug therapy OR TCM). Inclusion criteria (1): Clinical/experimental studies on DOP mechanisms or treatments (2); English-language articles; (3) Prioritization of meta-analyses (e.g., Vestergaard, Diabetologia 2005) and guidelines (IOF, 2018). Exclusion criteria: (1) Case reports; (2) Non-diabetic osteoporosis studies; (3) Articles without mechanistic/clinical outcomes. From 238 initial results, 170 references were retained based on relevance and impact. This review systematically examines the pathophysiological network of DOP, the mechanisms of action of glucose-lowering and anti-osteoporotic drugs, and recent advances in prevention and treatment. It aims to provide a theoretical foundation for optimizing clinical practice and guiding future research directions. This integrative review holds significant translational value for improving the prognosis of DOP patients and reducing the burden on healthcare systems.

## 2 Diabetes and osteoporosis

### 2.1 Diabetes mellitus

Diabetes mellitus (DM) represents a group of metabolic disorders characterized by chronic hyperglycemia, arising from a combination of diverse etiologies. The core pathological mechanisms involve insufficient insulin secretion and/or impaired insulin action. Based on distinct pathogenic pathways, DM is primarily classified into type 1 diabetes (T1D) and type 2 diabetes (T2D). T1D is characterized by autoimmune destruction of pancreatic β-cells, leading to an absolute deficiency of insulin, whereas T2D is predominantly marked by insulin resistance accompanied by relative insulin deficiency (17). As a critical global public health issue, diabetes not only exhibits severe metabolic dysregulation but also induces multi-system damage due to prolonged hyperglycemia, resulting in various chronic complications. With the acceleration of population aging, lifestyle changes, and dietary shifts, diabetes has emerged as the third most prevalent non-communicable disease, following cardiovascular diseases and malignancies. According to the latest statistics from the International Diabetes Federation (IDF), the global diabetic population reached 530 million in 2021 and is projected to exceed 780 million by 2045 (18).

### 2.2 Osteoporosis

Osteoporosis (OP) is a systemic skeletal disorder characterized by reduced bone mass and deterioration of bone microarchitecture. The core problem is disrupted balance between bone breakdown and formation, causing thinner trabecular and cortical bones, reduced strength, and higher fracture risk. This progressive degeneration of bone tissue structure predisposes individuals to fragility fractures (FF) even under low-energy trauma (e.g., falls from standing height) or during routine daily activities. As the most prevalent non-communicable skeletal disease worldwide, the prevalence of osteoporosis increases significantly with age. Epidemiological data indicate that the overall prevalence of OP in individuals aged 50 years and older is 19.2%, with a markedly higher prevalence in women (32.1%) compared to men (6.9%). Among those aged 65 and older, the prevalence rises to 32%, with women disproportionately affected at 51.6%, compared to 10.7% in men (19). Globally, osteoporosis is responsible for over 8.9 million fractures annually, equating to one osteoporotic fracture occurring every 3 seconds (20). This imposes a substantial burden on both individual health and socioeconomic systems.

### 2.3 Diabetic osteoporosis

Diabetic osteoporosis (DOP) is a significant long-term complication of diabetes mellitus, classified under secondary osteoporosis. It is pathologically characterized by diabetes-induced reductions in bone mass and microstructural damage, leading to increased bone fragility and elevated fracture risk. Studies have demonstrated that both type 1 and type 2 diabetes are significantly associated with an increased risk of fractures. While microvascular and macrovascular complications are the most commonly recognized sequelae of diabetes, osteoporosis and its associated fracture risk also warrant considerable attention in clinical practice (21). Globally, over 90,000 osteoporotic fractures annually are linked to DOP, posing a substantial threat to human health and socioeconomic development (20).

**Figure 1:**
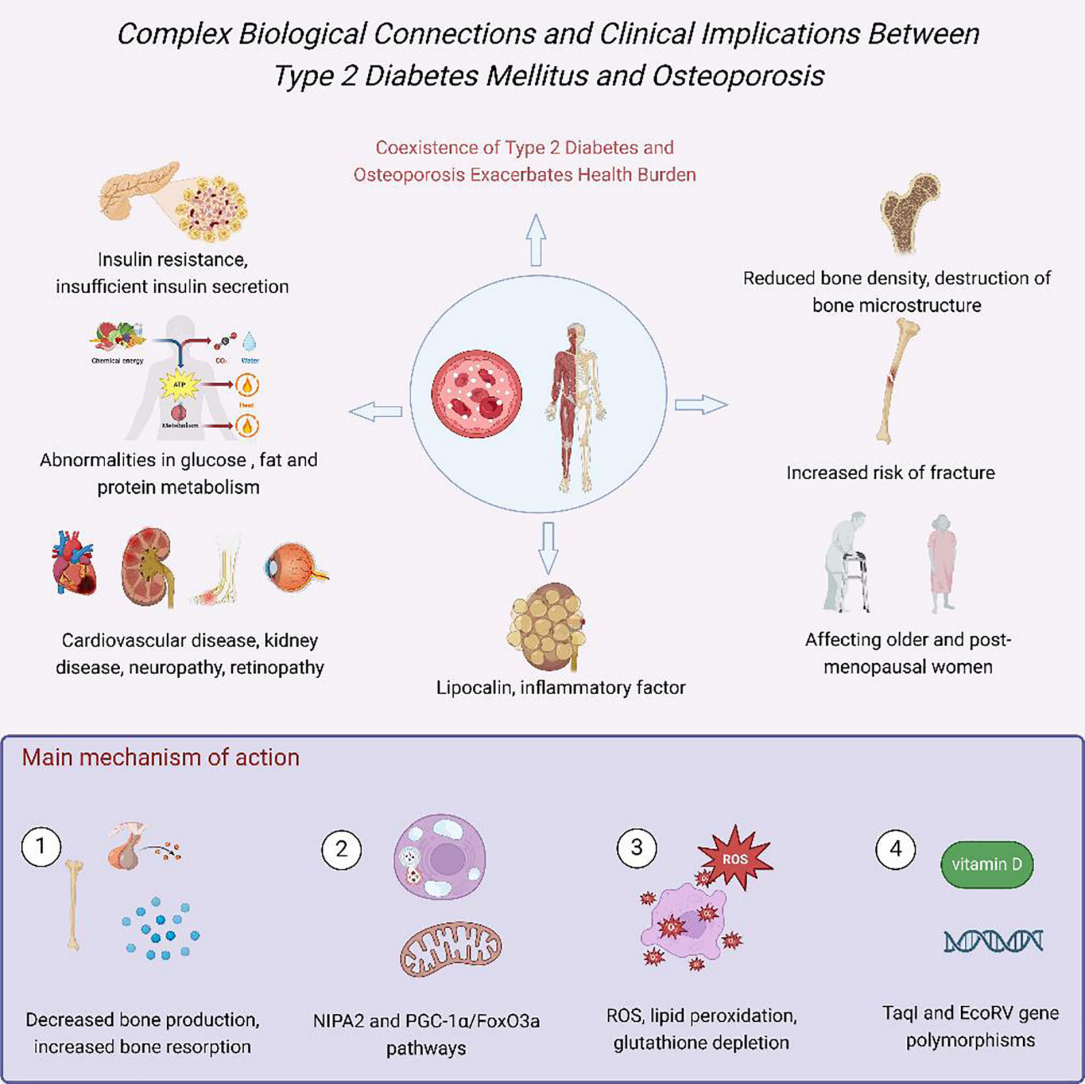
Complex biological connections and clinical implications between Type 2 Diabetes Mellitus (T2DM) and osteoporosis. Hyperglycemia, insulin resistance, and inflammation impair bone formation via AGEs, oxidative stress, and vascular dysfunction (25).

Compared to the general population, diabetic patients exhibit a significantly higher fracture risk. Research by Vestergaard et al. revealed that the risk of fractures at any site in diabetic patients is 0.5 – 2 times higher than in controls, with odds ratios (ORs) of 1.3 (95% CI 1.2 – 1.5) for type 1 diabetes and 1.2 (95% CI 1.1 – 1.3) for type 2 diabetes. For site-specific fractures, type 1 diabetic patients have a notably elevated risk of hip fractures (OR = 1.7, 95% CI 1.3 – 2.2), while type 2 diabetic patients show a relatively lower but still significant risk (OR = 1.4, 95% CI 1.2 – 1.6) (22). A meta-analysis by Fan et al. found that the pooled relative risk of hip fractures in diabetic patients compared to non-diabetic individuals was 2.07 (95% CI 1.83 – 2.33) (23). Further research by Wang et al. elucidated the relationship between diabetes and site-specific fracture risks: overall, diabetes was significantly associated with increased risks of hip (RR 1.77, 95% CI 1.56 – 2.02), upper arm (RR 1.47, 95% CI 1.02 – 2.10), and ankle fractures (RR 1.24, 95% CI 1.10 – 1.40), but not with distal forearm fractures (RR 1.02, 95% CI 0.88 – 1.19). Notably, type 1 diabetic patients exhibited significantly higher risks of total hip, hip joint, and ankle fractures compared to type 2 diabetic patients (P-values: 0.002, <0.001, and 0.029, respectively) (24). These findings underscore the strong association between diabetes and osteoporosis, as well as the critical importance of recognizing and addressing DOP in clinical management. **Figure 1** illustrated the intricate biological connections and clinical implications between Type 2 Diabetes Mellitus (T2DM) and osteoporosis.

## 3 Pathophysiological mechanisms of diabetic osteoporosis

### 3.1 Insulin deficiency

Insulin, a pivotal metabolic regulatory hormone, exerts extensive anabolic effects in the body, with its role in osteoblast anabolism being particularly significant. Studies have shown that insulin primarily regulates blood glucose levels through the PI3K/Akt signaling pathway while promoting cell proliferation and osteogenic differentiation (26). Insulin acts on osteoblasts through membrane receptors that detect insulin and trigger intracellular signaling pathways, starting a series of biological responses (27, 28). Specifically, insulin stimulates DNA synthesis, induces cell proliferation, and promotes the synthesis of osteocalcin and collagen, which are essential precursors for bone formation. Additionally, insulin upregulates the expression of the RUNX2 gene, a key regulator of bone metabolism that facilitates osteoblast differentiation and bone matrix maturation (29).

The critical role of insulin in bone metabolism provides a theoretical basis for understanding the differences in bone mineral density (BMD) between patients with type 1 diabetes (T1D) and type 2 diabetes (T2D). In T1D, the autoimmune destruction of pancreatic β-cells during early childhood leads to an absolute insulin deficiency, which persists throughout adolescence and results in insufficient bone mineralization. Clinical observations indicate that insulin therapy can partially improve bone mineralization in T1D patients (29). In contrast, bone metabolism in T2D patients is more complex: in the early stages of the disease, systemic insulin resistance accompanied by compensatory hyperinsulinemia may lead to increased bone mineralization. However, as the disease progresses to advanced stages, pancreatic β-cell function declines, insulin secretion decreases, and BMD significantly diminishes. This dynamic interplay highlights the central role of insulin in diabetic bone metabolism disorders and provides a crucial theoretical foundation for the prevention and treatment of diabetic osteoporosis (30, 31).

### 3.2 Insulin-like growth factor 1 (deficiency

Insulin-like growth factor 1 (IGF - 1), structurally similar to insulin, exerts significant anabolic effects on osteoblasts. Specifically, IGF - 1 acts on osteoprogenitor cells, stimulating DNA synthesis and promoting osteoblast differentiation, thereby significantly enhancing osteoblast activity. Additionally, IGF - 1 facilitates the formation of bone collagen while inhibiting its degradation, ultimately promoting bone matrix formation and mineralization. IGF - 1 is primarily synthesized and secreted by hepatocytes; however, in diabetic patients, chronic hyperglycemia suppresses the synthesis and release of IGF - 1, thereby impairing its osteogenic effects. Studies have shown that serum IGF - 1 levels are significantly reduced in diabetic patients, leading to decreased bone formation, which may represent a key pathological mechanism underlying diabetic osteoporosis (32). Further clinical research has confirmed that in patients with type 2 diabetes, serum IGF - 1 levels are positively correlated with bone mineral density (BMD), and reduced IGF - 1 levels are a significant risk factor for increased fracture risk in these individuals (32). Insulin and IGF - 1 deficiency directly impair osteoblast function, which explains the generally reduced BMD observed in T1DM patients and underscores the importance of early (e.g., as recommended by International Society for Pediatric and Adolescent Diabetes, ISPAD in late adolescence) and regular BMD screening (following IOF guideline) in these patients.

### 3.3 Hyperglycemic environment

A hyperglycemic environment exerts significant adverse effects on various tissues and cells. Studies have shown that elevated glucose levels activate the non-canonical Wnt/protein kinase C pathway and upregulate the expression of peroxisome proliferator-activated receptor gamma (PPARγ), thereby promoting adipogenesis and exacerbating bone loss (33). Hyperglycemia directly inhibits osteoblast/osteocyte activity via cytotoxic effects (34, 35). Specifically, osteoblasts exposed to high glucose environments demonstrate reduced proliferation, slowed extracellular matrix synthesis, and delayed maturation and mineralization processes (36). Furthermore, hyperglycemia increases osteoblast apoptosis and accelerates cellular senescence, further impairing their function. Under diabetic conditions, the osteocyte lacunar-canalicular system, constructed by osteoblasts, undergoes significant microstructural damage, characterized by abnormally enlarged lacunae, reduced canalicular network density, and disrupted cellular projections. These structural alterations lead to abnormal bone remodeling and altered mechanical properties, accelerating the progression of diabetic bone disease (36).

**Figure 2:**
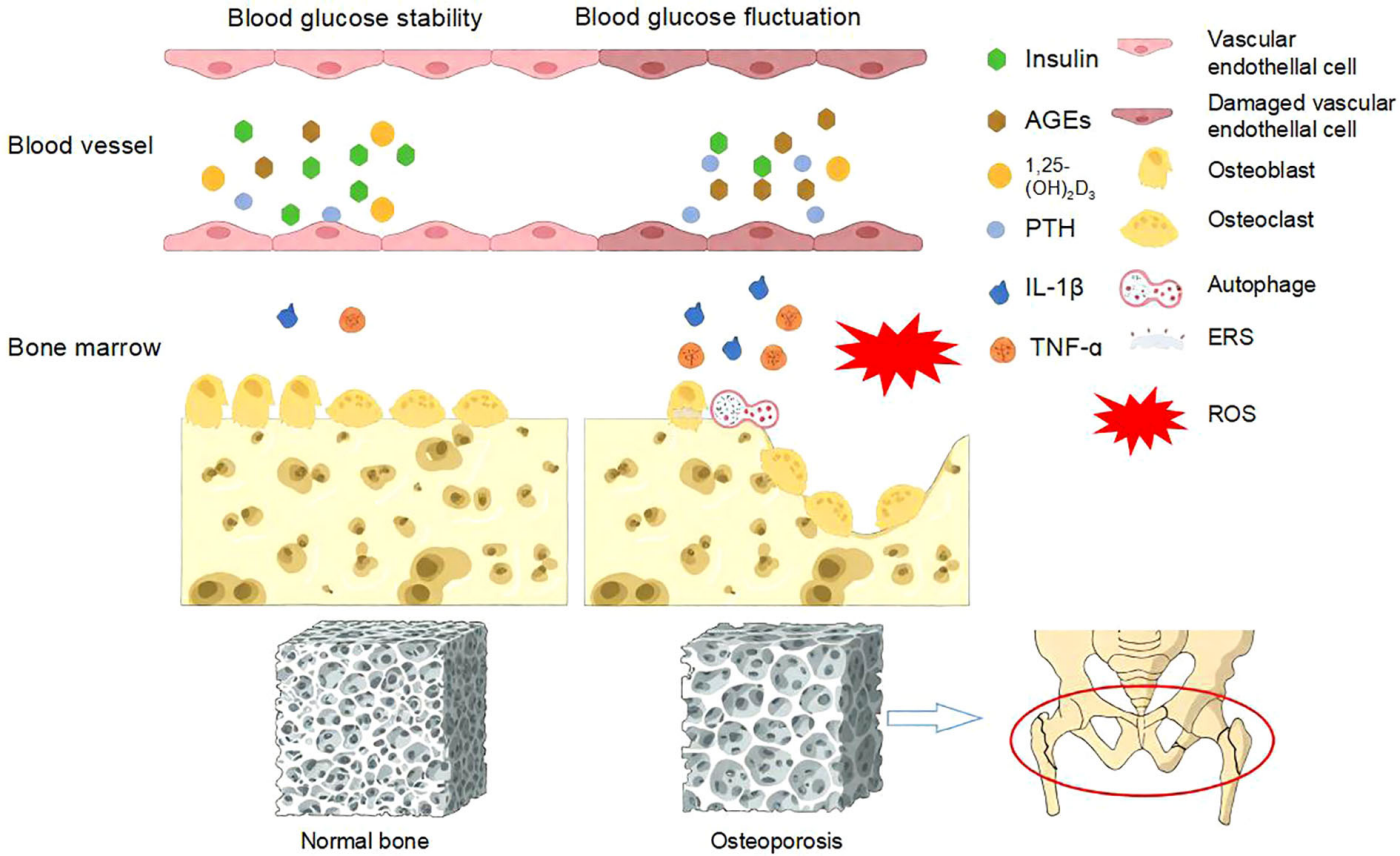
Pathogenesis of diabetic osteoporosis caused by blood glucose fluctuations. Blood glucose fluctuations can lead to oxidative stress, increased levels of advanced glycation end products and inflammatory mediators, reduced insulin secretion, decreased 1,25-(OH)_2_D_3_, elevated PTH levels, and the occurrence of ESR and autophagy. These factors, in turn, inhibit the function of osteoblasts and osteoclasts, leading to osteoporosis and osteoporotic fractures (38).

On the other hand, glucose, as a fundamental energy substrate, stimulates osteoclast activity, and the bone-resorbing function of osteoclasts is glucose concentration-dependent. Consequently, under hyperglycemic conditions, enhanced bone resorption coupled with impaired bone formation collectively contribute to rapid bone loss (37). **Figure 2** summarized the pathogenesis of diabetic osteoporosis induced by blood glucose fluctuations.

### 3.4 Accumulation of advanced glycation end products

Hyperglycemia may induce the formation of advanced glycation end products (AGEs) through non-enzymatic pathways. AGEs are generated via glycation reactions, which involve non-enzymatic interactions between ketones or aldehydes and protein amino groups, leading to protein dysfunction. This process occurs under both normoglycemic and hyperglycemic conditions, but its rate is influenced by substrate availability (39). Under normal physiological conditions, stable blood glucose levels result in a moderate rate of AGEs formation. However, in diabetic patients, significantly elevated glucose levels increase substrate availability, accelerating AGEs generation (39). Additionally, excess glucose can be converted to sorbitol via aldose reductase and subsequently to fructose by sorbitol dehydrogenase. Fructose and its metabolites, as potent glycating agents, further promote AGEs formation. AGEs exert multifaceted detrimental effects on the skeletal system, impairing extracellular matrix function and vascular structure.

#### 3.4.1 The key mechanisms by which AGEs impair bone quality

One critical mechanism by which AGEs compromise bone quality is through abnormal cross-linking with collagen in the bone matrix. This abnormal cross-linking reduces collagen elasticity, whereas enzymatic cross-linking is essential for maintaining bone strength by increasing collagen stiffness. Non-enzymatic cross-linking, however, is detrimental to bone quality (40, 41). The abnormal cross-linking of AGEs with collagen alters the structural and functional properties of proteins, leading to reduced overall bone strength and increased fragility (42). Due to the lower bone turnover rate in diabetic patients, proteins have more time to accumulate AGEs, resulting in significantly higher AGEs levels compared to non-diabetic individuals (43). Chronic hyperglycemia not only promotes glycation of the bone matrix but also impairs collagen turnover and matrix renewal, ultimately leading to impaired bone formation and increased skeletal fragility. Studies have shown that elevated AGEs content per gram of collagen disrupts the bone matrix and alters its material properties, making it more susceptible to mechanical failure under physiological stress levels. Studies show higher pentosidine levels in diabetic patients’ blood and urine predict greater fracture risk, regardless of bone density (44–46). This finding provides critical insights into the phenomenon of increased bone fragility in diabetic patients despite normal BMD.

3.4.2 The inhibitory effects of AGEs on osteoblasts.

Advanced glycation end products (AGEs) exert significant inhibitory effects on osteoblast function. Studies have demonstrated that AGEs suppress osteoblast differentiation and reduce the expression of alkaline phosphatase, thereby impairing osteogenic activity (47, 48). Sun et al. found that AGE-RAGE signaling blocks bone formation, while blocking RAGE boosts bone-related gene and protein expression (49). Boosting glyoxalase-1 (Glo-1) levels can counteract the harmful effects of AGE-RAGE signaling on bone marrow stem cells by detoxifying AGE precursors. The receptor for advanced glycation end products (RAGE), a multi-ligand receptor, binds to various ligands, including AGEs, Mac-1, high-mobility group box 1 (HMGB1), and S100 calcium-binding proteins (50– 51). *In vitro* experiments have shown that the binding of AGEs to RAGE activates multiple signaling pathways, including p38 mitogen-activated protein kinase (p38 MAPK), extracellular signal-regulated kinase 1/2 (ERK1/2), Janus kinase (JAK), and Rho-GTPases (52– 53).

Furthermore, AGEs significantly inhibit osteoblast proliferation, promote osteoblast apoptosis, and increase the expression of sclerostin, a negative regulator of bone formation, while reducing the levels of receptor activator of nuclear factor kappa-B ligand (RANKL) (54, 55). *In vitro* data indicate that high glucose levels and AGEs markedly upregulate sclerostin expression in osteocytes, further impairing bone formation (54). This preclinical observation has been validated in clinical studies, which show that individuals with prediabetes exhibit significantly higher sclerostin levels compared to controls, and these levels are closely associated with insulin resistance (56).Research by Ge et al. further highlights that AGEs significantly inhibit both the proliferation and differentiation of osteoblasts (57). Additionally, AGEs suppress the secretion of parathyroid hormone (PTH) (58), leading to inhibited bone turnover and a low bone turnover state, which exacerbates the development of diabetic osteoporosis. These findings not only elucidate the central role of AGEs in diabetic bone disease but also provide a critical theoretical foundation for the prevention and treatment of this condition. The accumulation of AGEs and their abnormal cross-linking is a key mechanism leading to decreased bone quality and the “BMD-fracture risk dissociation” phenomenon in T2DM patients. This suggests that relying solely on BMD and traditional FRAX scores may underestimate fracture risk in these patients. Therefore, clinical guidelines (such as the IOF position statement) strongly recommend including tools for assessing bone microstructure, like TBS, in the fracture risk evaluation system for T2DM patients. They also advise considering stricter BMD diagnostic thresholds (e.g., T-score ≤ -2.0).

### 3.5 Chronic inflammation

Diabetic patients often experience a state of chronic low-grade inflammation, characterized by the excessive activation of inflammatory cytokines, including tumor necrosis factor-alpha (TNF-α) and interleukin-6 (IL - 6). This chronic inflammatory state is not only closely associated with microvascular and macrovascular complications in type 2 diabetes (T2DM) but also exerts significant negative effects on bone remodeling processes (59, 60). Furthermore, a hyperglycemic environment promotes the differentiation of bone marrow mesenchymal stem cells (BMSCs) toward adipogenesis, accompanied by increased bone marrow fat deposition and the release of large amounts of free fatty acids and inflammatory cytokines (61). Among these inflammatory factors, TNF-α plays a pivotal role in promoting bone resorption. It stimulates the proliferation and differentiation of osteoclast precursor cells in the bone marrow into mature osteoclasts, thereby accelerating bone resorption (62). IL - 6, a multifunctional cytokine secreted by various human cells, not only promotes the division and proliferation of osteoclast precursor cells but also directly participates in the differentiation and maturation of osteoclasts. Additionally, IL - 6 enhances bone resorption by increasing the release of collagenase and the degradation of the bone matrix (63). These inflammatory cytokines also trigger intracellular oxidative stress, further exacerbating bone metabolism disorders. Chronic inflammation and oxidative stress not only promote bone resorption but may also affect bone formation markers. While bone turnover markers (BTMs) have limited value in predicting fracture risk in diabetic osteoporosis (DOP), their pattern changes (such as low osteocalcin levels) might provide supplementary information for understanding individual bone metabolic status. However, diagnosis and risk stratification still primarily rely on tools like BMD, FRAX, and TBS.

### 3.6 Oxidative stress

The homeostasis of the human internal environment relies on a dynamic balance between the oxidative system (primarily reactive oxygen species, ROS) and the antioxidant defense system (including superoxide dismutase, SOD), catalase, and glutathione peroxidase, GPx). Excessive ROS production plays a critical role in various physiological and pathological processes (64), such as causing oxidative damage to DNA, lipid membranes, and proteins, disrupting the antioxidant defense system, activating downstream pathways related to oxidative stress, and inducing indirect cellular damage (65, 66). Studies have shown that glycemic variability significantly elevates ROS levels. *In vitro* experiments demonstrate that compared to human umbilical vein endothelial cells (HUVECs) cultured under sustained hyperglycemic conditions, those exposed to intermittent hyperglycemia exhibit markedly increased ROS generation, leading to exacerbated cell apoptosis (67, 68). *In vivo* experiments by Horvath et al. investigated the effects of glycemic variability on oxidative stress in diabetic rats. The rats were divided into a stable glucose group (receiving long-acting insulin daily) and a glucose fluctuation group (receiving long-acting insulin every other day). After 14 days, the glucose fluctuation group showed significantly elevated nitrotyrosine levels and endothelial dysfunction compared to rats with stable or normal glucose levels (69). Regarding the impact of glycemic variability on bone cells, Zhang et al. found that glucose fluctuations lead to a rapid increase in ROS levels and induce oxidative stress, thereby impairing osteoblast function, inhibiting their activity and proliferation, and ultimately promoting osteoblast apoptosis (70).

### 3.7 Vascular damage

The hyperglycemic state in diabetes leads to both microvascular and macrovascular complications. Microvascular damage harms small blood vessels, causing poor circulation, weakened vessel function, blood vessel loss, reduced new vessel growth, and leaky vessels. Chronic hyperglycemia causes endothelial damage in microvessels, leading to vasomotor dysfunction, vascular rarefaction, and hemodynamic abnormalities, which significantly reduce blood perfusion to bones and bone marrow. The impaired endothelial barrier function exacerbates vascular leakage, resulting in bone marrow microenvironment disturbances (e.g., hemorrhage, edema) and further disrupting bone homeostasis. These changes directly affect the blood supply to bones, as skeletal health relies on adequate blood flow to deliver oxygen, nutrients, and hormones while removing metabolic waste (71). The microvascular system in bone marrow is critical for bone development, repair, and regeneration.

In diabetic patients, the bone marrow microenvironment undergoes significant alterations, characterized by reduced hematopoietic tissue and increased adipocyte accumulation (marrow adiposity). Vascular rarefaction and endothelial dysfunction contribute to insufficient bone perfusion, leading to hypoxia and oxidative stress. Oxidative stress damages endothelial cells and reduces the production of nitric oxide (NO), a key mediator of vasodilation. The diminished NO-mediated vasodilatory response, coupled with increased activity of vasoconstrictors such as endothelin-1, predisposes bone arteries to a constricted state (72). Diabetes also reduces key angiogenesis factors like VEGF and HIF - 1α, worsening new blood vessel formation and repair. Macrovascular complications, including atherosclerosis and peripheral artery disease, exacerbate these issues by reducing overall skeletal blood supply and worsening local microcirculatory dysfunction. Diabetes-related vascular damage leads to inadequate bone perfusion and hypoxia, impairing bone formation and repair capacity. This highlights the importance of controlling cardiovascular risk factors (such as hypertension and dyslipidemia) in DOP management, which not only aligns with diabetes care guidelines (e.g., ADA standards) but may also indirectly improve bone health outcomes.

## 4 Pathophysiological mechanisms of glucose-lowering agents in osteoporosis treatment

Glucose-lowering agents play a significant role in the treatment of diabetic osteoporosis (DOP), not only by effectively controlling blood glucose levels but also by influencing bone metabolism through various mechanisms. These diabetes medications are mainly classified by their mechanisms into: thiazolidinediones (TZDs), sulfonylureas, metformin, incretin-based drugs (GLP - 1 agonists and DPP - 4 inhibitors), SGLT2 inhibitors, and insulin. These drugs regulate bone formation and resorption through different signaling pathways, thereby exerting either positive or negative effects on bone health. **Table 1** provided a detailed summary of the functions and mechanisms of action (signaling pathways) of various glucose-lowering agents, offering important theoretical insights for the prevention and treatment of diabetic osteoporosis.

**Table 1:** Functions of glucose-lowering agents and mechanisms of action (signaling pathways) in the treatment of osteoporosis.

Drug/Category	Function	Mechanism of action (Signaling pathway)	References
Thiazolidinediones (TZDs) (e.g., rosiglitazone, pioglitazone)	Improve insulin sensitivity; adversely affect bone metabolism	Activate PPAR-γ, promoting adipogenesis over osteoblastogenesis; enhance osteoclastogenesis. Modulates FGF21 expression.	(73– 74)
Sulfonylureas (e.g., glimepiride)	Potential bone formation stimulation; increase fracture risk in elderly	Preclinical: Inhibits menopause-related skeletal changes (mechanism unclear).	(75– 76)
Metformin	Improve insulin sensitivity; promote osteoblast differentiation	Activates AMPK-dependent/independent pathways; inhibits PPAR-γ-mediated adipogenesis. Increases OPG and suppresses RANKL.	(22, 77– 78)
Incretin-Based Therapies (e.g., GLP - 1 agonists, DPP - 4 inhibitors)	Modulate bone turnover; prevent bone loss	GLP-1/GIP regulate bone homeostasis via unclear pathways. DPP - 4 inhibitors suppress bone resorption (partial clinical validation).	(79, 80)
SGLT2 Inhibitors (e.g., dapagliflozin, canagliflozin)	Exacerbate trabecular bone loss	Increase phosphate co-transport, stimulating PTH and bone resorption. Mechanism partially linked to renal glucose regulation.	(81, 82)
Insulin	No direct negative impact; higher fracture risk in T2D patients	Indirect risk via hypoglycemia-induced falls. No specific signaling pathway identified.	(83)

### 4.1 Thiazolidinediones

Thiazolidinediones (TZDs), represented by rosiglitazone and pioglitazone, are widely used antidiabetic drugs that primarily improve insulin sensitivity and reduce insulin resistance to achieve glycemic control. However, these drugs have significant adverse effects on bone metabolism. Studies have shown that TZDs impair osteoblast differentiation and reduce bone formation by modulating gene expression, leading to decreased bone mineral density (BMD) and increased fracture risk, particularly in female patients (73). Specifically, the use of rosiglitazone is associated with a 6 – 20% increase in bone resorption and a 4 – 13% reduction in bone formation markers in postmenopausal women, resulting in clinically significant bone loss (84). A pioglitazone meta-analysis reveals TZD treatment increases fracture risk in postmenopausal women, with longer treatment duration potentially heightening this risk.

The glucose-lowering effects of TZDs are primarily mediated through the activation of peroxisome proliferator-activated receptor gamma (PPAR-γ), which plays a critical role in glucose homeostasis and adipogenesis. PPAR-γ activation induces mesenchymal stem cells (MSCs) to differentiate into adipocytes rather than osteoblasts, thereby promoting adipogenesis at the expense of bone formation. This process disrupts skeletal metabolic balance. Additionally, PPAR-γ activation enhances osteoclastogenesis while suppressing osteoblastogenesis, leading to increased bone resorption and decreased bone formation (85– 86). TZDs also improve insulin resistance and sensitivity in diabetic patients by upregulating the expression of fibroblast growth factor 21 (FGF21), which enhances PPAR-γ transcriptional activity (87). However, excessive FGF21 expression contributes to bone loss. Research by Wei et al. demonstrates that rosiglitazone-induced bone loss can be prevented by inhibiting FGF21 function (74). Since TZDs are known to significantly increase bone loss and fracture risk (particularly in women), major osteoporosis and diabetes guidelines (including ADA, ESCEO, and AACE) all recommend avoiding or using these drugs with extreme caution in T2DM patients who already have osteoporosis or high fracture risk (as indicated by elevated FRAX assessment results).

### 4.2 Sulfonylureas

Sulfonylureas, a class of classic glucose-lowering agents, have shown inconsistent effects on bone metabolism in clinical studies. Current clinical data suggest that sulfonylureas do not significantly impact human bone health (75– 88). However, preclinical research indicates that certain sulfonylureas, particularly glimepiride, may have the potential to stimulate bone formation (89, 90). For instance, experimental data from ovariectomized rats demonstrate that glimepiride can inhibit menopause-related skeletal changes and promote bone formation. Despite these findings, sulfonylureas must be used cautiously due to their risk of inducing hypoglycemia, which is a major risk factor for fractures, especially in elderly and frail individuals (76, 91). A study on fracture risk in older men revealed that sulfonylurea users had a 66% higher risk of non-vertebral fractures compared to controls (91). Therefore, the use of sulfonylureas in elderly patients with osteoporosis requires careful consideration.

### 4.3 Metformin

Metformin, a classic oral hypoglycemic agent, not only improves insulin sensitivity to achieve glycemic control but also exhibits potential beneficial effects on bone health (22, 77). Preclinical studies have demonstrated that metformin promotes the differentiation of mesenchymal stem cells (MSCs) into osteoblasts while inhibiting adipogenesis and osteoclast differentiation (92– 93). Additionally, by reducing hepatic gluconeogenesis, metformin partially mitigates the negative impact of hyperglycemia on bone. Its mechanisms of action include both AMP-activated protein kinase (AMPK)-dependent and AMPK-independent pathways (94). Numerous *in vitro* studies have confirmed the osteogenic effects of metformin (95). Research by Zheng et al. further indicates that metformin regulates peroxisome proliferator-activated receptor gamma (PPAR-γ) through the AMPK pathway, thereby exerting protective effects on osteoblast differentiation and potentially counteracting osteoporosis (78).

Moreover, metformin inhibits osteoclast differentiation. Osteoblasts secrete receptor activator of nuclear factor kappa-B ligand (RANKL) and osteoprotegerin (OPG), which play critical roles in osteoclast differentiation. RANKL promotes osteoclast differentiation, while OPG, a decoy receptor for RANKL, inhibits this process. *In vivo* experiments have shown that metformin treatment increases OPG levels and suppresses RANKL expression, thereby reducing bone loss (96). However, not all studies support the positive effects of metformin on bone. For instance, research by Jeyabalan et al. found no significant impact of metformin on bone mass or fracture healing (97). Although clinical evidence for metformin’s bone-protective effects (Section 4.3) still requires confirmation through larger RCTs, its favorable preclinical data and relatively neutral clinical observations have made it one of the preferred insulin sensitizers for DOP patients (particularly those with fracture risk factors), and it is frequently recommended as first- or second-line therapy in clinical guidelines.

### 4.4 Incretin-based therapies

Incretin-based therapies, including glucagon-like peptide-1 (GLP - 1) receptor agonists and dipeptidyl peptidase-4 (DPP - 4) inhibitors, represent an important class of glucose-lowering agents. While GLP - 1 receptors are primarily expressed in pancreatic β-cells, their effects extend beyond glucose regulation. Studies suggest that GLP - 1 may modulate bone resorption through GLP - 2 and glucose-dependent insulinotropic polypeptide (GIP). In addition to promoting insulin secretion, GLP - 1 and GIP are involved in regulating bone turnover and maintaining bone homeostasis. Clinical studies have shown that treatments with exenatide and liraglutide effectively prevent bone loss associated with weight reduction, with liraglutide therapy increasing serum levels of total type I procollagen N-terminal propeptide (P1NP) by 16% (79, 80). However, findings on the impact of GLP - 1 agonists on fracture risk vary across clinical studies (98, 99), and no definitive conclusions have been reached. In mouse models, the DPP - 4 inhibitor sitagliptin has demonstrated inhibitory effects on bone resorption, a finding partially validated in clinical studies involving postmenopausal women (100, 101). Although some clinical studies suggest that DPP - 4 inhibitors may reduce fracture risk, a meta-analysis by Hidayat et al. did not support a significant association between DPP - 4 inhibitor use and fracture risk (102). GLP - 1 receptor agonists have demonstrated potential bone-protective effects, particularly during weight loss periods. Given their established cardiometabolic benefits, these agents may represent a preferred glucose-lowering option for DOP patients, especially those with high cardiovascular risk or obesity. However, current clinical guidelines have not yet specifically recommended them for bone protection purposes.

### 4.5 Sodium-glucose co-transporter-2 inhibitors

SGLT2 inhibitors represent a novel class of glucose-lowering agents. However, emerging evidence suggests that SGLT2 inhibitors may exacerbate trabecular bone loss (103). Although the precise mechanisms remain incompletely defined, this effect may be linked to the glucose-lowering mechanism of SGLT2 inhibitors. By reducing renal glucose reabsorption and sodium transport, SGLT2 inhibitors increase phosphate co-transport, which stimulates the parathyroid glands and enhances bone resorption (104). Treatment with dapagliflozin has been shown to elevate serum phosphate and parathyroid hormone (PTH) levels (105). In the Canagliflozin Cardiovascular Assessment Study (CANVAS), canagliflozin was associated with a higher incidence of fractures, primarily affecting the distal limbs, within just 12 weeks of treatment initiation compared to placebo (4.0% vs. 2.6%). For this reason, guidelines from organizations including ADA, EASD and ESCEO recommend cautious use of these medications in high fracture risk patients (such as elderly individuals, those with osteoporosis, fall history, or high FRAX scores), while prioritizing alternative glucose-lowering treatment options.

### 4.6 Insulin

As previously discussed, insulin plays a crucial role in bone metabolism, and insulin therapy itself is not considered to have a direct negative impact on bone health. However, some studies have reported that patients with type 2 diabetes receiving insulin therapy exhibit a higher risk of fractures compared to those not on insulin treatment (106). This observation may be influenced by the fact that patients requiring insulin typically have a longer disease duration and a higher prevalence of complications, which could independently contribute to increased fracture risk. Additionally, insulin-treated patients are at a higher risk of hypoglycemia, which may lead to an increased likelihood of falls and subsequent fractures (83). Current guidelines emphasize the need to optimize insulin regimens to reduce hypoglycemic episodes when treating elderly and osteoporotic patients, while also strengthening fall prevention measures (such as following the IOF six-step approach).

## 5 Pathophysiological mechanisms of anti-osteoporotic agents in osteoporosis treatment

The management of diabetic osteoporosis (DOP) should prioritize glycemic control, supplemented by anti-osteoporotic therapy (82). Insulin and glucose-lowering agents form the foundation of treatment for diabetic bone disease, but care must be taken to avoid medications with adverse skeletal effects, such as thiazolidinediones (TZDs). The pharmacological treatment of osteoporosis in diabetic patients is similar to that for other forms of osteoporosis and primarily includes agents that inhibit bone resorption, promote bone formation, and regulate bone metabolism. **Table 2** summarized the functions and mechanisms of action (signaling pathways) of anti-osteoporosis drugs.

**Table 2:** Functions and mechanisms of action (signaling pathways) of anti-osteoporosis drugs.

Drug/Category	Function	Mechanism of action (Signaling pathway)	References
Bone-Forming Agents			
Fluorides (e.g., sodium fluoride)	Stimulate osteoblast proliferation, inhibit osteoclast activity	Activates MAPK pathways; promotes mesenchymal stem cell differentiation into osteoblasts. Dual effects: low concentrations promote osteoblast activity, high concentrations induce toxicity.	(107– 108)
Teriparatide (PTH analog)	Increase bone mineral density (BMD), reduce vertebral fractures	Mimics human PTH activity; elevates bone formation markers (e.g., PINP).	(109)
Abaloparatide (PTHrP analog)	Promote bone formation	Comparable efficacy to teriparatide in preclinical models.	(110)
Simvastatin (Statin)	Promote bone formation, inhibit resorption	Inhibits RANKL-induced osteoclast formation via suppression of Akt, NF-κB, and MAPK pathways.	(111)
Androgens (e.g., testosterone)	Enhance bone protein synthesis, increase BMD	Promote osteocyte proliferation; regulate bone mass via androgen receptors.	(112)
SARMs (Selective androgen receptor modulators)	Treat glucocorticoid-induced osteoporosis	Activate androgen receptors with reduced side effects.	(113)
Strontium ranelate	Dual-action: inhibit resorption, promote formation	Modulates mesenchymal stem cells; enhances collagen synthesis. Reduces fracture risk via unclear pathways.	(114– 115)
Bone Resorption Inhibitors			
Calcitonin	Inhibit osteoclast-mediated bone resorption	Binds to calcitonin receptor (CTR), a GPCR; indirect effects on osteoblasts via osteoclast regulation.	(116, 117)
Bisphosphonates (e.g., Alendronate)	Suppress bone resorption	Promote osteoprotegerin (OPG) secretion; inhibit osteoclast activity via IL - 6 and TNF-α suppression.	(118, 119)
Raloxifene (SERM)	Reduce vertebral fractures, modulate bone turnover	Inhibits osteoclasts via selective estrogen receptor modulation.	(120, 121)
Estrogens	Increase bone mass, regulate bone homeostasis	Directly act on immune system, oxidative stress, and bone cells; reduce excessive bone turnover.	(122)
Bone Metabolism Modulators			
Calcium supplements	Maintain bone mineralization	Compensate calcium deficiency; support bone structure.	(123)
Active vitamin D (e.g., calcitriol)	Promote calcium absorption, bone mineralization	Binds to vitamin D receptors; reduces PTH synthesis; enhances calcium-phosphorus deposition.	(123)

### 5.1 Bone-forming agents

#### 5.1.1 Fluorides

Fluorides, such as sodium fluoride and disodium monofluorophosphate, are essential trace nutrients for bone growth. Fluoride directly stimulates osteoblast proliferation and inhibits osteoclast activity, thereby increasing bone mass (107). It also induces bone formation by promoting the differentiation of mesenchymal stem cells into osteoblasts (124). Fluoride shows dual bone effects: low doses inhibit osteoblast phosphatases, activating growth pathways that boost bone-forming cell activity. At high concentrations, fluoride exerts toxic effects on osteoblasts while inhibiting osteoclast activity (108).

#### 5.1.2 Parathyroid hormone analogs

Teriparatide, a recombinant human parathyroid hormone (33– 33) fragment, exhibits biological activity identical to human PTH. It significantly increases bone mineral density (BMD) in the lumbar spine and femoral neck over the long term and is an effective agent for reducing the risk of vertebral fractures in postmenopausal women with osteoporosis. Clinical trials have shown that serum levels of procollagen type I N-terminal propeptide (PINP), a marker of bone formation, increase consistently within three months of initiating teriparatide therapy (109). However, the high cost of teriparatide limits its widespread use. Abaloparatide, a novel parathyroid hormone-related protein (PTHrP) analog, has demonstrated comparable efficacy to teriparatide in mouse models at equivalent doses (110). Teriparatide represent potent therapeutic options for very high-risk patients (such as those with multiple vertebral fractures or extremely low BMD). While clinical data supporting their efficacy in DOP remains relatively limited but promising, current guidelines recommend their use in specific high-risk populations.

#### 5.1.3 Statins

Statins exhibit dose-dependent effects on bone metabolism: low doses increase bone resorption, while high doses promote bone formation. Simvastatin, for instance, demonstrates anabolic effects on bone by inhibiting osteoblast apoptosis and suppressing osteoclast differentiation and activity (125). Research by Chowdhury et al. revealed that simvastatin inhibits RANKL-induced osteoclast formation and function by suppressing the Akt, NF-κB, and MAPK signaling pathways, leading to downregulation of key transcription factors such as c-Fos and NFATc1 during osteoclastogenesis (111).

#### 5.1.4 Androgens and Prostaglandin E2

Androgens promote osteocyte proliferation, accelerate bone protein synthesis and mineralization, and increase trabecular bone volume and mass. Bone mineral density (BMD) in men is positively correlated with serum testosterone levels, which decline with age (112). Testosterone deficiency is a significant risk factor for male osteoporosis. Selective androgen receptor modulators (SARMs) have shown promising efficacy in treating glucocorticoid-induced osteoporosis in men (113). However, testosterone replacement therapy is associated with side effects such as acne and increased blood viscosity. Prostaglandin E2 (PGE2) is a potent bone anabolic agent that stimulates osteoblast differentiation and proliferation, promoting bone formation and increasing bone mass.

#### 5.1.5 Strontium salts

Strontium salts promote osteoblast proliferation and differentiation while inhibiting osteoclast formation and differentiation, inducing osteoclast apoptosis, and reducing bone resorption. They enhance bone mechanical strength without altering bone structure (114), making them dual-action bone metabolism regulators. Strontium also modulates mesenchymal stem cells, increases bone formation rates, and promotes the synthesis of collagen and non-collagenous proteins in the bone matrix, exerting anti-osteoporotic effects (126).

Strontium ranelate, a representative strontium salt, reduces the risk of osteoporosis-related fractures by simultaneously inhibiting bone resorption and promoting bone formation. A study by Reginster et al. demonstrated that strontium ranelate treatment reduced the relative risk of all non-vertebral fractures by 16% and major fragility fractures by 19% compared to placebo (115). However, the use of strontium salts is associated with increased risks of myocardial infarction, thromboembolic events, and rare severe skin reactions (DRESS syndrome), leading to their non-approval in the United States (103).

### 5.2 Bone resorption inhibitors

#### 5.2.1 Calcitonin

Calcitonin is a peptide hormone secreted by thyroid C cells and serves as a potent inhibitor of bone resorption. Its family peptide, amylin, can suppress osteoclast activity (127). The calcitonin receptor (CTR), a class B G protein-coupled receptor (GPCR), regulates calcium homeostasis and bone turnover upon activation by calcitonin, making it a therapeutic target for osteoporosis (116). Although calcitonin’s physiological role is to inhibit osteoclast-mediated bone resorption, its effects on osteoblasts are likely indirect and mediated through osteoclasts. Salmon calcitonin (MI), a peptide hormone with higher affinity for CTR than human calcitonin, is currently used as a clinical treatment for osteoporosis due to its ability to promote bone remodeling and inhibit bone resorption (117). However, calcitonin’s efficacy in fracture prevention is limited, and long-term use may increase the risk of cancer.

#### 5.2.2 Bisphosphonates

Bisphosphonates (BPs), stable analogs of pyrophosphate characterized by a P-C-P group, are first-line drugs for the prevention and treatment of osteoporosis in postmenopausal women and men, as well as for alleviating bone pain and fractures. BPs promote osteoblast secretion of osteoprotegerin (OPG), and their mechanism of action in inhibiting bone resorption is twofold. On one hand, they regulate osteoclast proliferation, differentiation, and apoptosis; on the other hand, they suppress bone resorption by inhibiting osteoclast-mediated expression of cytokines such as interleukin-6 (IL - 6) and tumor necrosis factor-alpha (TNF-α) (118). Representative BPs include alendronate, zoledronate, and risedronate. While BPs are highly effective in treating osteoporosis, they are associated with side effects such as atypical femoral fractures and osteonecrosis of the jaw. For patients with severe osteoporosis and high fracture risk, long-term fracture prevention and bone mineral density (BMD) restoration may be challenging with BPs alone. A sequential approach—starting with bone-forming agents like teriparatide followed by long-term anti-resorptive therapy—may be beneficial (119).

#### 5.2.3 Estrogens

Osteoporosis is closely associated with estrogen levels. Estrogens regulate bone homeostasis by directly acting on the immune system, oxidative stress, and bone cells, increasing bone mass, effectively modulating excessive bone turnover, and improving bone mineral density (BMD) (122). Raloxifene, the first selective estrogen receptor modulator (SERM) approved by the FDA for postmenopausal osteoporosis, is primarily used in postmenopausal women with significantly reduced estrogen levels. Raloxifene inhibits osteoclast activity through multiple signaling pathways without increasing the risk of endometrial cancer (120). It effectively reduces the risk of vertebral fractures in osteoporotic patients (121). Currently, most clinical treatments for postmenopausal osteoporosis are based on estrogen replacement therapy. However, long-term estrogen use increases the risk of breast, cervical, and endometrial cancers in women.

### 5.3 Bone metabolism modulators (active vitamin d and calcium supplements)

Calcium is a primary component of human bones and is essential for maintaining bone mineralization and metabolism. Osteoporosis is largely caused by calcium deficiency, which can be addressed by supplementing with calcium carbonate, calcium citrate, or calcium gluconate to increase calcium intake. Vitamin D promotes the absorption and storage of calcium ions in the body and directly influences calcium-phosphorus balance. Vitamin D metabolizes calcitriol, a circulating calcium-regulating hormone, accelerates gene synthesis in osteoblasts, and promotes bone mineralization (123). Active vitamin D directly acts on the parathyroid glands, reducing the synthesis and secretion of parathyroid hormone (PTH). It also binds to vitamin D receptors in osteoblasts and osteoclasts, ultimately promoting calcium and phosphorus deposition and mineralization in bones. However, severe adverse effects, such as hypercalcemia-induced renal dysfunction, may occur, necessitating regular monitoring of blood calcium levels.

### 5.4 Traditional Chinese medicine for osteoporosis treatment

Although currently approved anti-osteoporotic drugs can reduce the risk of fragility fractures, their long-term use is often limited by safety concerns, significant side effects, and low target specificity. In contrast, TCM, with its minimal side effects, offers unique advantages in the treatment of chronic diseases, including osteoporosis. TCM has a long history of use in preventing and treating osteoporosis (128). Based on modern medical understanding of osteoporosis pathology and clinical manifestations, TCM classifies osteoporosis as “bone impediment”, “bone wilting”, or “bone withering”. The primary pathogenesis involves kidney deficiency, blood stasis, and dual deficiency of qi and yin, with the disease located in the bones. The essence of osteoporosis in TCM is “kidney essence deficiency and spleen deficiency leading to malnourishment,” characterized by blood stasis obstruction (129). **Table 3** summarized the active ingredients and mechanisms of action (signaling pathways) of commonly used traditional Chinese medicines.

**Table 3:** Active components of commonly used traditional Chinese medicines and their mechanisms of action (signaling pathways).

Herbal medicine	Active component(s)	Mechanism of action (Signaling pathway)	References
Epimedium	Icariin (flavonoid glycoside)	Modulates JNK/c-Jun, Wnt/β-catenin, and Notch signaling pathways; enhances osteogenic activity.	(130– 131)
Eucommia ulmoides	Leaf extract	Regulates Wnt/β-catenin signaling pathway; reduces RANKL-induced bone resorption-related gene expression.	(132)
Salvia miltiorrhiza	Salvianolic acid, Tanshinones	Reduces bone formation impairment via KLF15/PPARγ2 pathway; inhibits osteoclast differentiation.	(133, 134)
Drynaria fortunei	–	Modulates osteoblast and osteoclast activity; improves glucocorticoid-induced osteoporosis.	(135)
Achyranthes bidentata	Polysaccharides	Promotes bone formation; potential anti-osteoporotic agent.	(135)
Dipsacus asper	Swertiamarin (iridoid glycoside)	Activates p38 signaling pathway via membrane estrogen receptor-α and GPR30.	(136)
Psoralea corylifolia	Isoflavonoids, Corylifol A, Psoralen	Activates ER-Wnt-β-catenin pathway; inhibits ROS via Nrf2; increases osteogenic markers (Runx2, ALP).	(137– 138)
Cistanche deserticola	Polysaccharides, Cistanoside A	Inhibits RANKL/RANK-induced NF-κB and PI3K/AKT pathways; downregulates TRAF6.	(139– 140)
Cordyceps sinensis	Polysaccharides, peptides, isoflavonoids	Reduces blood calcium/phosphorus levels; improves femoral neck strength in osteoporosis models.	(141, 142)

According to TCM theory, bone health is closely related to kidney function. Kidney deficiency affects calcium and phosphorus metabolism, leading to reduced bone mineral density and impaired bone metabolism. Many kidney-nourishing herbs have been found to restore bone health and are used to treat bone-related diseases. TCM treatment primarily employs methods to tonify the kidneys and strengthen bones, focusing on replenishing kidney essence, while also supporting liver and spleen health, promoting blood circulation, and alleviating pain (143).

Herbal formulations, with their multi-component and multi-target advantages, have gained significant attention for the treatment of chronic diseases. Herbal medicines are derived from various parts of medicinal plants (e.g., leaves, stems, buds, flowers, or roots) and sometimes include non-plant components (e.g., insects, deer antlers, snakes, shells, and fossil powders). These ingredients can be used in their raw form or as water or alcohol extracts (144). They contain multiple active compounds, such as flavonoids, polysaccharides, saponins, and alkaloids, which exert therapeutic effects by regulating hormone levels, calcium-phosphorus metabolism, inflammatory responses, osteoclast activity, and osteoblast proliferation, ultimately improving bone microstructure.

Flavonoids, as the primary active components of many herbal medicines, play a significant role in treating osteoporosis. *Epimedium*, known for its kidney-tonifying and bone-strengthening properties, has a long history of use in treating bone diseases in China. Studies have shown that *Epimedium*, with its high flavonoid content, can effectively treat osteoporotic distal radius fractures (130). Icariin, the main active flavonoid glycoside in *Epimedium*, enhances osteogenic activity by modulating the JNK/c-Jun, Wnt/β-catenin, and Notch signaling pathways (131, 145).

*Eucommia ulmoides* leaf extract promotes osteogenic differentiation of bone marrow mesenchymal stem cells (BMSCs) by regulating the Wnt/β-catenin signaling pathway and reducing RANKL-induced bone resorption-related gene expression (132). *Salvia miltiorrhiza* has also been used historically to treat bone diseases (146). With advancements in modern analytical techniques, numerous compounds have been isolated from *Salvia miltiorrhiza*. Salvianolic acid, one of these compounds, plays a role in reducing bone formation impairment through the KLF15/PPARγ2 signaling pathway (133). Additionally, tanshinones have been shown to inhibit osteoclast differentiation and are considered potential candidates for osteoporosis treatment (134).

*Drynaria fortunei* improves glucocorticoid-induced osteoporosis by modulating osteoblast and osteoclast activity. *Achyranthes bidentata*, another traditional Chinese herb for osteoporosis, contains polysaccharides that have been extensively studied for their ability to promote bone formation, making them potential agents in anti-osteoporotic therapy (135).

Swertiamarin, a major active iridoid glycoside isolated from *Dipsacus asper*, exerts anti-osteoporotic effects by interacting with membrane estrogen receptor-α and GPR30, thereby activating the p38 signaling pathway (136). Asperosaponin VI, a triterpenoid saponin, exhibits anti-osteoclastic activity by inhibiting RANKL-induced osteoclast differentiation and function (147).

Certain compounds in *Psoralea corylifolia* exhibit anti-osteoporotic (OP) activity by activating the ER-Wnt-β-catenin signaling pathway, with isoflavonoids being the most potent (137). Corylifol A, a representative flavonoid, reduces reactive oxygen species (ROS) production by activating Nrf2, thereby inhibiting osteoclast formation and activation (148). Psoralen, another flavonoid from *Psoralea corylifolia*, increases the expression of osteogenic markers such as Runt-related transcription factor 2 (Runx2), osterix, type I collagen (Col1), and alkaline phosphatase (ALP), making it a targeted therapy for osteoblast-mediated OP (138).

*Cistanche deserticola*, an edible medicinal herb, inhibits RANKL/RANK-induced activation of downstream NF-κB and PI3K/AKT pathways and blocks the activity of key osteoclastogenic proteins, NFAT2 and c-Fos (139). Polysaccharides from *Cistanche deserticola* reduce RANKL-mediated ROS production in osteoclasts, impairing osteoclastogenesis and bone resorption (149). Cistanoside A, a phenylethanoid glycoside isolated from *Cistanche deserticola*, has potential in treating OP by downregulating TRAF6 (140).

*Cordyceps sinensis* contains active components such as polysaccharides, cordyceps peptides, isoflavonoids, amino acids, and alcohols. *Cordyceps sinensis* and its extracts have shown therapeutic effects on various types of osteoporosis. In a classic bilateral ovariectomy rat model simulating postmenopausal osteoporosis, Wei Qi et al. demonstrated that daily oral administration of 10 ml of *Cordyceps sinensis* mycelium extract significantly reduced blood calcium, blood phosphorus, and urinary calcium levels while increasing the mechanical strength of the femoral neck, effectively improving osteoporosis (141, 142).

In summary, many TCM formulations and their derived compounds demonstrate potential therapeutic effects in preventing and treating OP. Additionally, TCM therapies such as acupoint application, acupuncture, and massage can be used to improve bone mineral density.

## 6 Prevention and treatment of diabetic osteoporosis

The diagnosis of diabetic osteoporosis requires a comprehensive evaluation using multiple methods. Currently, clinical diagnostic techniques include dual-energy X-ray absorptiometry (DEXA), the Fracture Risk Assessment Tool (FRAX), trabecular bone score (TBS), and high-resolution peripheral quantitative computed tomography (HR-pQCT). DEXA, the gold standard for osteoporosis diagnosis, reveals that patients with type 1 diabetes (T1DM) exhibit significantly reduced bone mineral density (BMD), while those with type 2 diabetes (T2DM) show BMD values 5 – 10% higher than non-diabetic individuals (150). This paradoxical phenomenon may be related to the increased bone fragility in T2DM patients due to impaired bone quality. Despite higher BMD, T2DM patients exhibit elevated fracture risk due to trabecular microarchitecture deterioration, marrow adiposity, and AGEs-induced collagen brittleness. While FRAX includes T1DM as an osteoporosis risk factor, it substantially underestimates fracture risk in T2DM patients by 30 - 50%, according to studies (151). Therefore, a stricter diagnostic threshold (-2.0 vs. -2.5) is recommended for T2DM patients (152). Notably, FRAX assessments for T2DM patients require multi-parameter adjustments, including incorporating TBS to evaluate bone microstructure, adding rheumatoid arthritis as an input, reducing the femoral neck T-score by 0.5, and increasing the age parameter by 10 years (153). To more accurately assess fracture risk in diabetic patients, the combined use of FRAX and TBS is considered a more effective approach. By incorporating TBS as an adjustment factor for FRAX, clinicians can achieve more precise fracture risk stratification in diabetic patients, thereby better guiding decisions regarding osteoporosis treatment initiation (154). For example, a study conducted in Manitoba, Canada, compared four different methods for improving FRAX performance in type 2 diabetic patients (153). The results demonstrated that FRAX adjusted by TBS provided a more accurate fracture risk assessment in this population. Furthermore, TBS can identify elevated fracture risk in diabetic patients who have normal BMD but impaired bone microarchitecture. These patients might otherwise be overlooked if assessed solely using FRAX without TBS adjustment (155).

Advanced imaging techniques, such as TBS, analyze the spatial variation of grayscale in DEXA images to assess trabecular bone microstructure. A TBS value ≤1.2 indicates significant microstructural degradation and serves as a critical supplementary parameter for FRAX evaluation in T2DM patients. HR-pQCT provides three-dimensional volumetric BMD data, revealing that T1DM patients exhibit significant reductions in trabecular and cortical bone volume, closely associated with glycemic control and microvascular complications. In contrast, T2DM patients primarily show increased cortical bone porosity. Histomorphometric analysis demonstrates that even T1DM patients with good glycemic control exhibit normal bone formation markers but have significantly elevated abnormal deposition of advanced glycation end products (AGEs) in bone collagen. This non-enzymatic cross-linking, also observed in T2DM patients, may reduce bone matrix elasticity and increase fragility.

Bone is a dynamic tissue that undergoes continuous remodeling through bone formation (mediated by osteoblasts) and bone resorption (mediated by osteoclasts) (156). Bone turnover markers (BTMs) are biochemical byproducts released into blood or urine during these processes, reflecting the rate and balance of bone remodeling (157). In DOP patients, bone metabolism is often dysregulated. Notably, individuals with T2DM may exhibit normal or even elevated BMD while still having increased fracture risk-a phenomenon that cannot be explained by BMD measurements alone. This highlights the need for more nuanced indicators of bone health, and BTMs provide precisely such dynamic information. P1NP (procollagen type I N-terminal propeptide), a byproduct of type I collagen synthesis which is the primary structural protein of bone matrix, reflects osteoblastic activity in both healthy postmenopausal women and those with T2DM (158). Studies show reduced P1NP levels in diabetic patients. Osteocalcin, a non-collagenous protein secreted by osteoblasts that is critical for bone matrix mineralization, exists in an undercarboxylated form (ucOC) that may regulate glucose metabolism (158). This protein serves as a potential link between bone and glucose homeostasis, and its levels are consistently observed to be lower in diabetic populations. The combined evaluation of BTMs such as P1NP and osteocalcin with BMD and clinical tools like FRAX+TBS could enable earlier detection of skeletal fragility in diabetes, particularly in cases where BMD alone is misleading (159). The integrated application of these diagnostic tools enables a more accurate assessment of bone quality and fracture risk in diabetic patients, providing a critical foundation for individualized prevention and treatment strategies.

The International Osteoporosis Foundation (IOF) provides professional guidelines for the diagnosis and management of diabetic osteoporosis (160). For patients with type 2 diabetes (T2DM), baseline bone mineral density (BMD) testing using dual-energy X-ray absorptiometry (DEXA) is recommended five years after diagnosis, provided there are no additional risk factors (e.g., glucocorticoid use or advanced age). If the initial assessment indicates a low fracture risk, follow-up evaluations are advised every 3 – 5 years (160). For individuals with type 1 diabetes (T1DM), the International Society for Pediatric and Adolescent Diabetes (ISPAD) recommends BMD screening during late adolescence, particularly for those with celiac disease, poor long-term glycemic control, or existing microvascular complications.

In terms of prevention and management, lifestyle modifications to maintain a normal body mass index (BMI) are considered crucial. Overweight or obese patients should aim to reduce weight through calorie control and increased physical activity. Additionally, the IOF has proposed a six-step guide for fall prevention, which includes ensuring a safe home environment (e.g., adequate lighting and handrails), wearing slip-resistant footwear, correcting visual impairments, and maintaining healthy exercise and dietary habits. Diabetic patients should incorporate foods rich in vitamins, minerals, and protein into their daily diet. Diabetic patients should incorporate foods rich in vitamins, minerals, and protein into their daily diet to support bone health. Vitamin D levels should be maintained at ≥20 ng/mL. To achieve this, patients are advised to take 1000 – 2000 IU of vitamin D daily. If daily dietary calcium intake falls below 1.2 g, calcium supplements should be prescribed to ensure adequate bone mineralization and reduce fracture risk.A meta-analysis suggests that a Mediterranean diet based on fresh fruits, vegetables, and fish can reduce the risk of fractures and microvascular complications in T2DM patients (161).

## 7 Future perspectives

With the deepening understanding of the pathological mechanisms underlying diabetic osteoporosis (DOP), future research should focus on two major directions: the development of novel drugs and the systematic exploration of multi-dimensional interaction networks.

### 7.1 Development of novel antidiabetic drugs

While current glucose-lowering agents effectively improve glycemic control, their dual effects on bone metabolism require optimization. Emerging antidiabetic drugs, such as GLP - 1 receptor agonists and SGLT2 inhibitors, have demonstrated potential to enhance bone quality by modulating energy metabolism, inflammatory pathways, or oxidative stress. For instance, GLP - 1 receptor agonists may promote bone formation and inhibit bone resorption through AMPK signaling activation, while SGLT2 inhibitors could indirectly influence bone metabolism balance via the kidney-bone axis. Future studies should further elucidate the bone-protective mechanisms of these drugs and develop novel agents that combine glucose-lowering and bone-targeting functions, such as gut hormone-based peptide analogs or small-molecule compounds targeting bone microenvironment metabolic reprogramming. Additionally, the development of novel bone therapeutics targeting specific defects in bone quality—such as AGEs-RAGE pathway inhibitors, ferroptosis modulators, or mitochondrial function regulators—holds promise for overcoming the limitations of traditional anti-osteoporotic drugs and achieving precise intervention in diabetic bone disease.

### 7.2 Personalized medicine approaches

The heterogeneity of diabetic osteoporosis (DOP) demands a paradigm shift toward precision medicine, integrating molecular profiling, biomarker stratification, and tailored therapeutic interventions to optimize skeletal outcomes. Emerging evidence supports the use of genomic risk prediction, where polymorphisms in VDR (e.g., TaqI, EcoRV), RANKL/RANK/OPG signaling, and FOXQ1 expression patterns may identify patients at heightened risk for AGEs-mediated bone fragility or poor response to conventional therapies. Combining these genetic markers with clinical variables-such as diabetes duration, glycemic variability, and microvascular complications-could refine fracture risk assessment beyond traditional tools like FRAX, particularly in T2DM patients with preserved BMD but impaired bone quality. Biomarker-guided strategies hold promise for individualized treatment selection, leveraging serum pentosidine to quantify advanced glycation end-product burden or sclerostin levels to assess Wnt pathway suppression. Patients exhibiting low bone turnover (evidenced by depressed osteocalcin/P1NP ratios) may benefit preferentially from anabolic agents like teriparatide, while those with elevated resorption markers could derive greater protection from antiresorptives such as bisphosphonates or denosumab.

### 7.3 Systematic analysis of drug interaction networks and multi-target intervention strategies

Current research predominantly focuses on the mechanisms of individual drug classes, while the synergistic or antagonistic effects of combined “glucose-lowering and bone-protective” therapies in clinical practice remain poorly understood. Future efforts should systematically dissect the interaction networks between antidiabetic and anti-osteoporotic drugs at molecular, cellular, and systemic levels. For example, metformin and bisphosphonates may synergistically inhibit bone resorption through autophagy pathway regulation, whereas thiazolidinediones (TZDs) and RANKL inhibitors may exhibit reduced efficacy due to PPARγ signaling interference. Further exploration of key regulatory nodes, such as ferroptosis, VDR signaling, and Wnt/β-catenin pathways, will reveal the mechanisms underlying combined therapy efficacy and potential risks. Moreover, integrated multi-omics and artificial intelligence-based analyses can identify multi-target intervention strategies, such as dual-functional molecules that simultaneously inhibit AGEs accumulation and enhance bone formation, or multi-dimensional approaches targeting the bone-vascular-immune axis. These advancements will not only provide a theoretical foundation for optimizing clinical combination therapies but also pave the way for minimizing adverse drug reactions and developing personalized treatment regimens.

## 8 Conclusions

The prevention and treatment of diabetic osteoporosis (DOP) require a comprehensive approach based on its multifactorial pathological mechanisms, integrating metabolic regulation, bone microenvironment homeostasis, and systemic inflammation. This review systematically summarizes the pathophysiological network of DOP, encompassing core mechanisms such as insulin/IGF-1 deficiency, advanced glycation end products (AGEs) accumulation, oxidative stress, and vascular damage. It also elucidates the dual effects and potential risks of glucose-lowering and anti-osteoporotic drugs. Although current diagnostic and therapeutic approaches have significantly improved patient outcomes, precise interventions targeting bone quality deterioration remain challenging. Future research should focus on exploring novel drug targets, optimizing combination therapies, and advancing personalized treatment strategies through translational medicine. Ultimately, the goal is to achieve dual metabolic and skeletal benefits in diabetic bone disease.
